# Planning an integrated disease surveillance and response system: a matrix of skills and activities

**DOI:** 10.1186/1741-7015-5-24

**Published:** 2007-08-15

**Authors:** Helen N Perry, Sharon M McDonnell, Wondimagegnehu Alemu, Peter Nsubuga, Stella Chungong, Mac W Otten, Paul S Lusamba-dikassa, Stephen B Thacker

**Affiliations:** 1 Centers for Disease Control and Prevention, Atlanta, GA, USA; 2 Dartmouth Medical School, Hanover, NH, USA; 3 World Health Organization, Regional Office for Africa, Brazzaville, Republic of the Congo; 4 World Health Organization, Geneva, Switzerland; 5 World Health Organization, Regional Office for Africa, Brazzaville, Republic of the Congo

## Abstract

**Background:**

The threat of a global influenza pandemic and the adoption of the World Health Organization (WHO) International Health Regulations (2005) highlight the value of well-coordinated, functional disease surveillance systems. The resulting demand for timely information challenges public health leaders to design, develop and implement efficient, flexible and comprehensive systems that integrate staff, resources, and information systems to conduct infectious disease surveillance and response. To understand what resources an integrated disease surveillance and response system would require, we analyzed surveillance requirements for 19 priority infectious diseases targeted for an integrated disease surveillance and response strategy in the WHO African region.

**Methods:**

We conducted a systematic task analysis to identify and standardize surveillance objectives, surveillance case definitions, action thresholds, and recommendations for 19 priority infectious diseases. We grouped the findings according to surveillance and response functions and related them to community, health facility, district, national and international levels.

**Results:**

The outcome of our analysis is a matrix of generic skills and activities essential for an integrated system. We documented how planners used the matrix to assist in finding gaps in current systems, prioritizing plans of action, clarifying indicators for monitoring progress, and developing instructional goals for applied epidemiology and in-service training programs.

**Conclusion:**

The matrix for Integrated Disease Surveillance and Response (IDSR) in the African region made clear the linkage between public health surveillance functions and participation across all levels of national health systems. The matrix framework is adaptable to requirements for new programs and strategies. This framework makes explicit the essential tasks and activities that are required for strengthening or expanding existing surveillance systems that will be able to adapt to current and emerging public health threats.

## Background

Effective and timely public health responses depend upon the ability of health systems to provide reliable and timely information for action [1]. The global smallpox and polio eradication programs provide examples of the critical role that surveillance plays in linking surveillance data to targeted public health responses [2-4]. The value of surveillance today is also evident in the World Health Organization (WHO) call for influenza surveillance for early detection of human disease caused by a potential pandemic strain [5]. There are WHO recommendations that detail what countries need to do to prepare for pandemic influenza and that urge countries to invest their own resources to improve their national capacities for surveillance and response [5,6]. However, in many countries surveillance resources are scarce except for selected high priority diseases. Consequently, improvements in surveillance are usually limited to well-funded categorical disease programs. As a result, surveillance systems lack the flexibility to respond to emerging threats such as pandemic influenza.

How, then, should countries proceed to streamline their resources to strengthen their national surveillance systems? The WHO Recommended Surveillance Standards (2000) suggest how multiple levels of the health system (peripheral, intermediate and central levels) can be organized into a comprehensive surveillance system [7]. While WHO surveillance guidelines for single-disease programs prescribe components inherent to each single disease control program, there is limited guidance available for countries that want to integrate multiple surveillance systems and reform existing structures to meet requirements for improved health information. Experience in designing and implementing the integrated disease surveillance and response (IDSR) strategy in the African region might provide insights to those public health leaders trying to respond to the demands for timely information about public health events.

### Integrated disease surveillance in the African region

During the 1990s, there was an increase in the number of severe outbreaks of meningococcal disease, cholera, viral hemorrhagic fevers and measles as well as expansion of disease across national borders [8-12]. In October 1996, national governments affected by these outbreaks met with officials from WHO and its partners in Burkina Faso to develop action plans to improve capacities to respond to epidemics. Following this meeting, the World Health Organization Regional Office for Africa (WHO-AFRO) developed and implemented district level training for epidemic preparedness and response for four epidemic-prone diseases: cholera, measles, meningococcal disease and yellow fever [13].

Building upon this effort to improve epidemic preparedness and response in the African region, the 48th WHO Regional Committee for Africa met in September 1998 in Zimbabwe and adopted a strategy called Integrated Disease Surveillance and Response (IDSR). IDSR aims to "create functional IDSR systems in African countries that will generate information for timely action thus contributing to the reduction of mortality, disability and morbidity" [14]. The WHO-AFRO IDSR strategy focuses on the district level, but the goal of the IDSR strategy is to develop sufficient surveillance and response capacities at each level of the national system so that a flexible national infectious disease surveillance system will result [1,14,15]. In a national adaptation of the strategy, a country might choose to focus initially on a few diseases depending on national resources and capacities [14]. A country where IDSR is functional would:

1. use standard IDSR case definitions to identify and report priority diseases;

2. collect and use surveillance data to alert higher levels and trigger local action;

3. investigate and confirm suspected outbreaks or public health events using laboratory confirmation when indicated;

4. analyze and interpret data collected in outbreak investigations and data from routine monitoring of other priority diseases;

5. use information from the data analysis to implement an appropriate response;

6. provide feedback within and across levels of the health system; and

7. evaluate and improve the performance of surveillance and response systems [14-16].

**Figure 1:**
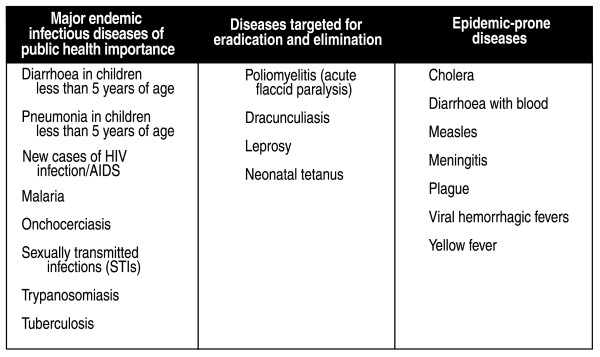
Priority infectious diseases for WHO-AFRO integrated disease surveillance and response strategy.

The strategy targeted 19 priority communicable diseases that are divided into three categories: epidemic-prone diseases, diseases targeted for eradication and elimination, and diseases that are endemic (Figure 1). These diseases were targeted because they remain the leading causes of illness, death and disability in the African region, and because well-known, effective responses for their prevention and control already exist [14]. A directive from WHO-AFRO added pandemic influenza to IDSR and recommended establishment of a focal point for influenza at the national level [6].

**Figure 2:**
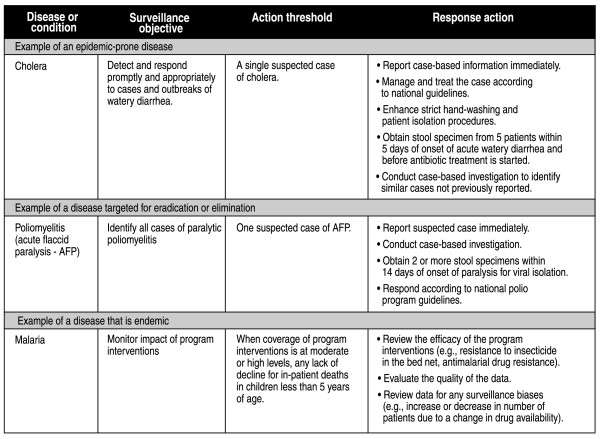
Examples of action thresholds.

The IDSR strategy is based on the surveillance threshold approach used for detecting outbreaks in disease control programs for smallpox, yellow fever, meningococcal disease and polio [3,18,19]. Action thresholds are set for each disease, and the range of recommended actions and activities that the thresholds should trigger is specified according to national policy (Figure 2). For example, a single suspected case of yellow fever is the threshold for conducting an outbreak investigation. Surveillance and laboratory data collected during the investigation are linked to appropriate, relevant response actions such as mass immunization campaigns, improved case management, and community education. In the case of an endemic disease (such as malaria) with a moderate or high level of coverage for its disease control intervention, a lack of decline in deaths is the threshold for reviewing the intervention and taking action to improve detection and response capabilities.

Between 2000 and 2002, WHO-AFRO and the United States Centers for Disease Control and Prevention (CDC) collaborated in the development of district level technical guidelines for implementing the IDSR strategy. The collaborators were asked to determine how the specific components of a multilevel, multi-disease surveillance and response system could be integrated at the district level to promote the public's health [1,17,21]. This paper describes a method to transform multiple, complex surveillance frameworks into an integrated public health surveillance system linked with existing disease control programs to implement a timely response.

## Methods

Using document reviews, semi-structured interviews with key informants, and discussions with officials from specific disease programs, we conducted a task analysis to define the surveillance skills and activities required for implementing surveillance recommendations for each of 19 priority diseases targeted by IDSR. "Skills" were defined as the actions of individual health workers and "activities" as an outcome of the combined skills of one or more health workers. A five-step task analysis enabled reconciliation of several complex surveillance components into a set of skills-based, observable actions [22,23]. The steps used are outlined below.

### Step 1: specify the surveillance and response requirements for each priority disease or condition targeted by IDSR

We reviewed the standard practice guidelines for each specific disease involved in the IDSR strategy to identify the surveillance requirements (for example, standard case definitions, data elements for reporting, thresholds and laboratory testing, and response actions) for each of the 19 priority diseases. We consulted disease experts to confirm and modify our understanding of surveillance and response requirements for each disease. When our search revealed gaps or variations in technical elements, an international technical collaboration team comprised of WHO, CDC and other epidemiologists, disease control experts, laboratory chiefs, and program managers was asked to help standardize the descriptions of surveillance and response activities across disease categories. Their comments were aggregated and common areas of agreement were found. This step resulted in standard surveillance case definitions for both the community and district levels, definitions of surveillance action thresholds for timely public health actions, clarification of the role of laboratory confirmation in suspected outbreaks, and specification of minimum data elements for reporting and analysis.

### Step 2: identify the skills and activities that are common to each specific disease and categorize the features within seven core functions – case identification or detection, reporting, analysis, investigation, response, feedback and program evaluation

After we achieved agreement on disease-specific requirements (for example, consistent wording of case definitions), we sorted the recommendations according to surveillance functions. We included laboratory activities within the seven core functions, positioning laboratory support as integral to a public health surveillance system.

### Step 3: choose a visual representation of the multi-level, multi-disease system

**Figure 3:**
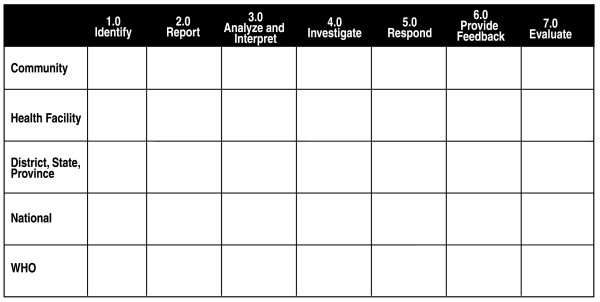
Matrix components.

We selected a matrix format to display the skills and activities selected in step 2 (Figure 3).

### Step 4: relate the skills in disease-specific systems with a core surveillance and response function in a multi-level system

Core surveillance and response functions are those activities for detection of cases and patients, registration of cases in log books and registers, confirmation with laboratory results, analysis of reported data, use and feedback of data, and epidemic preparedness and response [24]. Associated support functions that enable implementation of the core surveillance and response activities include coordination, supervision, training, and mobilization of resources [24].

The column headings listed across the top of the matrix (Identify, Report, Analyze, Interpret, Investigate, Respond, Provide Feedback and Evaluate) incorporate both surveillance and support functions. The levels of the health system – community (typically a village), health facilities, district or intermediate (such as a state or province), national and international (WHO country and regional offices) – were displayed as row headings (see Additional file 1). Each cell in the matrix was a prompt for deciding on the placement of the skills and activities derived in Step 2 [22]. For example, we described dissemination of standard case definitions throughout a national system as a responsibility related to each level in the "Identify" column. This makes explicit the role of the national level to establish standard case definitions and action thresholds. The role of the district or intermediate level is to disseminate the standard case definitions through training, supervision and monitoring. The health facility uses the definitions to identify the cases or outbreaks of the priority diseases or conditions. Simplified case definitions could be used locally to link the community to the health facility and, eventually, to other levels. At all levels it is important to adapt existing systems to local needs.

### Step 5: cross-check the assignment of skills and activities to specific functions or levels

To validate the assignment of surveillance skills and activities to specific functions, we conducted multiple review sessions to obtain further feedback and confirmation from disease program and surveillance experts in WHO, CDC, and other public health organizations. One outcome from this step was an observation that the placement of a skill at any one of the levels depended upon the availability of resources and policies that support an individual system. We portrayed this observation with a broken line between rows (representing the levels) to indicate flexibility during adaptation to national contexts and resources. The challenges affecting the placement of the skill at a particular level might be financial (such as when funding limits resources to infrastructure for a single, vertical disease program) but can also be technical (such as lack of skill to inoculate cholera specimens properly into transport media) and cultural (such as reluctance to collect spinal fluid specimens in a meningitis epidemic). This external validation has led to adoption of practical solutions such as more effective resource acquisition, provision of laboratory training kits, and use of culturally sensitive community education.

## Results

The results of our study were both the production of the matrix (see Additional file 1) and documentation of its use as a planning tool. We wanted to document how the matrix can assist health staff in seeing the relationship between what they do at sub-national levels and how well the national system performs overall. Examples of effective use of the matrix as a planning tool for nations to identify practical steps towards system integration are given below.

### Clarifying roles and responsibilities during implementation of IDSR: Tanzania

During 2002, IDSR technical and funding partners working in Tanzania (i.e. the Ministry of Health, WHO Tanzania, USAID Tanzania, CDC, and a contractor) needed to clarify roles and responsibilities for multiple partner and national program managers working with the Ministry of Health to implement IDSR. Using the matrix, planners were able to ensure that activities within all the cells of the matrix were accounted for in their overall plan and that each activity had a champion. In a separate exercise, a program implementation team used the matrix to clarify the job descriptions for sub-national staff charged with carrying out IDSR activities in the Tanzanian system [25]. The matrix exercise enabled the partners to specify accountability for each function at each level of the health system.

### Prioritizing activities in a multi-year plan of action: Uganda

In 2001, the Uganda IDSR plan of action was developed based on the results of an assessment of the surveillance system. While the matrix represents a complete system, each column or row provides a framework for identifying priorities within particular focus areas. For example, the priority for the first year of the plan of action was to adopt and disseminate standard case definitions throughout the system, thereby addressing skills within the first column of the matrix. In this way, essential features would be in place in order to ensure success with the following year's plans for improving completeness of reporting, provision of feedback, and training in the analysis and use of data at all levels [26].

### Developing instructional goals for applied field epidemiology training programs: Central America

CDC worked with its partners in Central America (Ministries of Health, USAID, and national universities) to develop a regional training program for applied field epidemiology during 2002. Curriculum planners from the national programs met in Guatemala with consultants from applied epidemiology training programs in other countries to develop curriculum plans for teaching surveillance systems (C. Sanchez-Vargas, personal communication, 2002). The participants used the matrix to develop instructional goals and objectives for teaching the principles and practices of public health surveillance by focusing on surveillance functions rather than disease-specific systems. Each year, the matrix is used as a tool for teaching the organization of surveillance in the introductory course in the regional applied epidemiology training program for Central America and Hispañiola.

### Clarifying perceptions and objectives to revise a system: Philippines

Program managers in the Philippines wanted to integrate a routine reportable disease system within a large sentinel disease system situated in selected health facilities throughout the country. Program managers in the Philippines Department of Health representing the health information systems, infectious disease programs, and the IDSR program worked with their partners to use the matrix format to compare where essential surveillance activities took place in an integrated system. The product was a focused analysis of each system's characteristics and specification of exact actions that health staff would need to do at each level to meet the goals of a revised system. This analysis based on the IDSR matrix assisted the Philippines Department of Health in development of a systematic plan for human resource planning and development, computer system support, and a list of priority diseases (unpublished report, CDC, 2002)

### Developing skills-based guidance and public health training for district level staff: Africa

In 2002, WHO and CDC completed the first draft of the district level *Technical guidelines for IDSR in the African region *[17]. The organization of the guidelines was based on the matrix. Adaptation of these guidelines is a required step in the WHO-AFRO strategy for implementing IDSR in national systems [14]. The matrix is included in the guidelines so that it can be adapted to meet national priorities. By December 2006, 41 of the 46 countries in the African region had adapted the technical guidelines to meet their own public health priorities and situations. The guidelines are also linked to the development of core indicators for measuring progress of IDSR and the implementation of national plans of action for improving their existing public health surveillance systems.

The skills and actions on the matrix were the basis for developing learning objectives for a district level training course developed by WHO-AFRO in 2002–2003. The course is structured according to surveillance functions on the matrix. The training materials incorporate the skills and practical steps described in the IDSR district level technical guidelines. As of December 2006, the WHO-AFRO IDSR training course had been adapted and implemented in 41 countries.

## Discussion

During the course of development of the IDSR matrix, several key principles about surveillance systems were reinforced, namely: the functions of detection, analysis, investigation, response, feedback and evaluation are interdependent and should always be linked; an effective system at each level requires participation from the levels above and below; the core elements that comprise a successful surveillance system must all be present and performed well, or the risk of failure increases for achieving the surveillance and control objectives; an integrated system might minimize delay in taking public health action; and system success depends on people who have the appropriate skills for their assigned tasks.

This last principle underlies the need for well-trained health officers and staff who are the key to ensuring successful use of information for action in the IDSR initiative. While this is a common understanding, many health workers at sub-national levels lack sufficient public health surveillance skills [23]. Often, health staff whose primary duties are other than public health must carry out surveillance tasks required by national policies. These same health staff must also provide patient care, manage health facilities and comply with multiple reporting tasks for several disease specific activities. The result is that data are often not accurate, complete or timely [27]. Inefficiencies also result when health staff must meet the objectives of numerous competing surveillance systems. This result is felt at higher levels when public health leaders do not have basic data about urgent and critical events [28]. Consequently, decision-makers lack relevant and timely surveillance data that can inform decisions about actions to control and prevent public health threats.

The IDSR matrix establishes a skills-based vision of integrated surveillance and response with practical applications for public health leaders, program managers, and other decision makers tasked with creating an integrated system. The matrix approach has helped planning teams clarify perceptions about existing surveillance and response systems and assist with conceptualizing new ones. The matrix presentation encourages discussion among constituents of the value of surveillance to evidence-based decision-making in general, rather than focusing solely on single skill sets for a particular vertical system. Its presentation also makes clear the skills and activities that must be in place for achieving the desired outcomes and the need for human resources in achieving objectives set forth in new initiatives. Finally, the matrix suggests how disease-specific financial resources could be streamlined with objectives to improve national surveillance and response infrastructures.

We have seen the matrix perform as a powerful adjunct to public health curriculum developers for defining the learning objectives and relevant skills for designing, integrating, and maintaining surveillance systems that are tightly linked with disease control programs. In training epidemiologists and other health program managers to lead and build functional, integrated systems, we must include not only how to evaluate surveillance systems but also how to implement and support them with skills for planning, managing and integrating these systems.

As a result of the global direction for early detection of emerging public health threats such as pandemic influenza, there is increased demand from high-level policy makers to demonstrate results with disease control resources and to be informed about disease events in advance of the media or competing interests. Countries must also respond to the call to implement WHO International Health Regulations (2005), and meet the Millennium Development Goals. These demands illuminate the practical concern for better trained and more health staff and more responsive health systems. Public health leaders who must respond to this demand need accessible and clear tools for rapid implementation of public health interventions and strategies that simplify the organization of multiple and complex systems with single, integrated systems. When implementing new initiatives, the activities, skills, and resources necessary for successful performance are not always defined. By making the skills and activities explicit, public health leaders and program developers can produce objective-based criteria and clear expectations for successful outcomes. Attention can then be focused on realistic target setting, training, supervision, resource mobilization, monitoring and evaluation.

We believe the IDSR matrix contributes to our ability to create integrated systems that meet the needs of policy makers and improve the community wellbeing. The usefulness of the matrix warrants further evaluation in other settings including the analysis of costs as well as benefits.

## Conclusion

The successful adaptation of the IDSR matrix and district level guidelines in the African region suggests to other nations the utility of a practical, skill-based approach to developing and building functional surveillance and response systems. Technical partners and ministries of health working in Africa have been able to use the skill-based approach to develop indicators for measuring progress based on specific objectives and outcomes described in the matrix. Most importantly, the tools and procedures developed through the IDSR strategy encourage use of local data by district level health management and epidemic preparedness teams to define, monitor, and respond to public health problems in their own communities and contribute to the achievement of national and international goals for disease prevention and control.

## Competing interests

The author(s) declare that they have no competing interests.

## Authors' contributions

HP conceived and carried out the study and drafted the manuscript. SM conceived and carried out the study. WA, PN, SC, MO, PL, and ST each participated in the design, analysis and coordination of the study. All authors read and approved the final manuscript.

## Pre-publication history

The pre-publication history for this paper can be accessed here:



## Supplementary Material

Additional file 1 Detect and respond to priority diseases matrix Click here for file
