# Supplementary material for: Planning an integrated disease surveillance and response system: a matrix of skills and activities
Source: BMC Med. 2007 Aug 15;5:24. doi: 10.1186/1741-7015-5-24 (PMC1988797; doi:10.1186/1741-7015-5-24)
Supplement: Additional file 1 — Detect and respond to priority diseases matrix [file 1741-7015-5-24-S1.pdf]

*Figure 4: Detect and Respond to Priority Diseases Matrix*

# DETECT AND RESPOND TO PRIORITY DISEASES

World Health Organization, Regional Office for Africa, Centers for Disease Control and Prevention Technical guidelines for integrated disease surveillance and response in the African region Atlanta, Georgia: Public Health Service, Centers for Disease Control and Prevention/Epidemiology Program Office, Division of International Health; National Center for Infectious Diseases, Division of Bacterial and Mycotic Diseases. July 2001:10-11

| | 1.0 Identify<br>*Note: Laboratory steps apply to each level with access to laboratory services* | 2.0 Report | 3.0 Analyze and Interpret | 4.0 Investigate<br>*Note: These steps assume appropriate laboratory capacity* | 5.0 Respond | 6.0 Provide Feedback | 7.0 Evaluate and Improve the System |
|---|---|---|---|---|---|---|---|
| **Community** | • Use simple case definitions to identify priority diseases or conditions in the community | • Know which health events to report to the health facility and when to report them | • Involve local leaders in observing and interpreting disease patterns and trends in the community | • Support case investigation activities such as informing the community of the problem, case finding, collecting of specimens and other activities | • Assist health authorities in selecting response activities<br>• Participate in response activities<br>• Mobilize community resources appropriate for response activity<br>• Carry out community health education | • Give feedback to community members about reported cases and prevention activities | • Decide if public health action took place as planned<br>• Evaluate the community response to the public health action |
| **Health Facility** | • Use standard case definitions to identify priority diseases or conditions that present in:<br>-inpatient and outpatient services<br>-community reports<br>-private sector reports<br>• Record information about suspected cases in clinic register and patient charts<br>• *Use local laboratory capacity to diagnose suspected cases*<br>• *Use standard protocols to process laboratory specimens*<br>• *Collect and transport clinical specimens for laboratory evaluation* | • Report case-based information for immediately notifiable diseases<br>• Report data gathered from inpatient and outpatient services and from community and private sector sources<br>• Report summary data to next level<br>• *Report laboratory results from screening sentinel populations at target sites (for example, STI clinic, MCH service, blood bank)* | • Prepare and periodically update graphs, tables and charts to describe time, person, and place for reported diseases and conditions<br>• Identify and report immediately any disease or condition that:<br>- exceeds an action threshold<br>- occurs in locations where it was previously absent<br>- occurs more often in a population group than previously<br>- presents unusual trends or patterns<br>• Interpret results. Discuss possible public health action with district team<br>• *Observe changes in trends during routine analysis of laboratory results* | • Take part in investigation of reported outbreaks<br>• Collect, package, store and transport specimens for laboratory testing<br>• Use investigation and laboratory results to confirm the outbreak<br>• *Process and record laboratory results*<br>• *Provide the results to clinical staff and patients* | • Treat cases and contacts according to standard case management guidelines<br>• Use appropriate infection control measures<br>• Carry out public health response with the district level<br>• Mobilize community involvement in the response<br>• Advocate for resources | • Give feedback to community members about outcome of reported cases and prevention activities | • Monitor timeliness and completeness for reporting routine and case-based information to the district level<br>• Evaluate routine detection and reporting of priority diseases and conditions<br>• Evaluate preparedness for and timeliness of response activities<br>• Evaluate appropriateness of case management<br>• Take action to improve reporting practices<br>• Take action to improve readiness for timely response to outbreaks<br>• Maintain contact with community to maintain preparedness and prevention activities<br>• *Monitor the interval between receipt of specimens and sending of results*<br>• *Monitor quality of laboratory results* |
| **District, State, Province** | • Maintain activities for collecting routine surveillance data in a timely way<br>• Review records of suspected outbreaks<br>• *Collect and transport clinical specimens for laboratory evaluation* | • Support health facilities in knowledge and use of standard case definitions for reporting priority diseases and conditions<br>• Make sure health facility staff know when and how to report priority diseases and conditions<br>• Promptly report immediately notifiable diseases to the next level<br>• *Report laboratory results to national and local officials* | • Define denominators and obtain data for ensuring accurate denominators<br>• Aggregate data from health facility reports<br>• Analyze case-based data by person, place and time<br>• Calculate rates and thresholds<br>• Compare current data with previous periods<br>• Prepare and periodically update graphs, tables and charts to describe time, person and place for reported diseases and conditions<br>• Make conclusions about trends, thresholds, and analysis results<br>• Describe risk factors for priority disease or conditions | • Arrange and lead investigation of reported cases or outbreaks<br>• Assist health facility in safe collection, packaging, storage and transport of laboratory specimens for confirmatory testing<br>• Receive and interpret laboratory results<br>• Decide if the reported outbreak is confirmed<br>• Report the confirmed outbreak to the next level<br>• *Distribute specimen collection kits for special surveillance activities* | • Select and implement appropriate public health response (for example, depending on the disease, plan to strengthen case management, conduct immunization activity, improve control and prevention activities)<br>• Convene epidemic response committee and plan response<br>• Conduct training for emergency activities<br>• Plan timely community information and education activities<br>• Alert nearby areas and districts about the confirmed outbreak | • Alert nearby areas and districts about outbreaks<br>• Give health facilities regular, periodic feedback about routine control and prevention activities | • Monitor and evaluate program targets and indicators for measuring quality of the surveillance system<br>• Monitor and evaluate timeliness and completeness of reporting from health facilities in the district<br>• Monitor and evaluate timeliness of response to outbreaks<br>• Monitor routine prevention activities and modify them as needed |
| **National** | • Establish steps for surveillance of sentinel populations<br>• Conduct special surveys to gather information about reported cases, outbreaks or unusual events<br>• Define and update surveillance needs and implement training for and other support to each level<br>• Advocate for adequate resources to support the identification and reporting of cases<br>• *Set policies and procedures with national reference laboratory*<br>• *Use national reference laboratory for maintaining quality control and standards* | • Set policies and procedures for reporting priority diseases and conditions at each level<br>• Include private sector laboratories in the reporting network<br>• Support reporting activities throughout the system | • Set policies and procedures for analyzing and interpreting data<br>• Aggregate data received from district reports<br>• Make sure each level uses appropriate denominators for analysis<br>• Interpret trends from national perspective<br>• Adapt or define action thresholds<br>• Provide training resources for analyzing and interpreting data<br>• Analyze data for time, person and place<br>• Analyze map and stratify by district and other factors<br>• Make conclusions based on analysis results<br>• Provide reports and share data with national authorities and WHO as required<br>• Define public health analysis skills appropriate to each level of personnel in the system | • Alert laboratory and support its confirmation activities: supplies, transport media, logistics, transport of specimens<br>• Support activities for investigating reported outbreaks: supplies, logistics, equipment, budget<br>• Collaborate with international authorities as needed during investigations<br>• Notify regional, international networks about confirmed outbreak<br>• *Process specimens from investigation and send timely results as required to each level*<br>• *Request additional specimens as needed*<br>• *Take part in epidemic response team* | • Set policies and procedures for responding to cases and outbreaks of priority diseases and conditions<br>• Support epidemic response and preparedness activities<br>• Report and disseminate results of outbreak response in bulletins, media, press releases and briefings | • Give feedback about response activities to each level<br>• Give districts regular, periodic feedback about routine control and prevention activities<br>• Develop and periodically distribute regional bulletin for epidemiology and public health | • Establish and disseminate policies and procedures for monitoring surveillance and outbreak response activities<br>• Establish policies and practices for supervising surveillance and outbreak response activities<br>• Evaluate detection and reporting activities, and make improvements as needed:<br>- Monitor and evaluate program targets and indicators for measuring quality of the surveillance system<br>- Monitor and evaluate timeliness and completeness of reporting from intermediate levels<br>- Monitor and evaluate timeliness of national support for outbreak response<br>- Monitor and evaluate effectiveness of district-level outbreak response activities<br>• Monitor routine prevention activities and modify as needed<br>• Monitor quality assurance for laboratories at lower levels |
| **National WHO Representative, WHO Regional Office** | • Support policy setting at national and regional level for detecting priority diseases<br>• Mobilize resources for training, logistics and supervision<br>• Develop and distribute standard guidelines for surveillance "best practices"<br>• Inform countries about problems that may cross borders or have impact on regional areas | • Receive reports of outbreaks and international notifiable diseases | • Establish and disseminate standard guidelines for analysis of data for each priority disease | • Communicate recommendations for case investigation and laboratory confirmation<br>• Mobilize resources for improving laboratory capacity and skills<br>• Mobilize resources for investigation and confirmation as required, based on national level need and request<br>• Provide laboratory training and equipment<br>• Establish guidelines for preparedness and outbreak investigations<br>• Participate in investigations as requested | • Support response activities (technical experts, guidelines)<br>• Report to and inform international authorities about outbreak response<br>• Calculate response indicators and report status to next level<br>• Assist national level with epidemiological response and development of public health action | • Provide feedback for collaboration with national and regional levels<br>• Inform countries about problems that may cross borders or have impact on regional levels<br>• Report analysis results in regional and international bulletins for disease trends and patterns<br>• Develop and distribute regional bulletin for epidemiology and public health | • Use reports from countries to measure their systems and advocate for improvements |

July, 2000
